# A 2‐month post‐COVID‐19 follow‐up study on patients with dyspnea

**DOI:** 10.1002/hsr2.435

**Published:** 2021-11-17

**Authors:** Md. Khairul Islam, Mohammad Faisal Hossain, Md. Maruf Ahmed Molla, Md. Mohiuddin Sharif, Pratyay Hasan, Fahima Sharmin Hossain, Ayesha Sikder, Md Gias Uddin, Md. Robed Amin

**Affiliations:** ^1^ Department of Medicine Dhaka Medical College Hospital Dhaka Bangladesh; ^2^ Department of Pharmaceutical Sciences Appalachian College of Pharmacy Oakwood Virginia USA; ^3^ Department of Virology National Institute of Laboratory Medicine and Referral Center Dhaka Bangladesh; ^4^ Department of Medicine National Institute of Mental Health and Hospital Dhaka Bangladesh; ^5^ Respiratory Care Highlands ARH Medical Centre Prestonsburg Kentucky USA; ^6^ Non‐communicable Disease Control Directorate General of Health Services Dhaka Bangladesh

**Keywords:** asthma, COPD, D‐dimer, dyspnea, post‐COVID‐19, serum ferritin, smoking

## Abstract

**Background and aims:**

Dyspnea is one of the most common symptoms associated with the COVID‐19 caused by novel coronavirus SARS‐CoV‐2. This study aimed to assess the prevalence of dyspnea, observe co‐variables, and find predictors of dyspnea after 2 months of recovery from COVID‐19.

**Methods:**

A total of 377 patients were included in the study based on their responses and clinical findings during initial admission to the hospital with COVID‐19. After excluding five deceased patients, a total of 327 patients were interviewed through telephone using a 12‐point dyspnea scale and using relevant questions to gauge the patient clinically. Interviews were carried out by trained physicians, and responses were recorded and stored. All analyses were carried out using the statistical programming language R.

**Results:**

Of the total 327 participants in the study, 34% had stated that they were suffering from respiratory symptoms even after 2 months of COVID‐19. The study demonstrated that patient oxygen saturation level SpO_2_ (*P* = .03), D‐dimer (*P* = .001), serum ferritin (*P* = .006), and the presence and severity of dyspnea are significantly correlated. In addition to that, patient smoking history (*P* = .012) and comorbidities such as chronic obstructive pulmonary disease (COPD) (*P* = .021) were found to be statistically significant among groups.

**Conclusion:**

These findings of this study can be useful for predicting and managing long‐term complications of COVID‐19.

## 1 INTRODUCTION

The virus responsible for COVID‐19 disease is a strain of coronavirus, much like its predecessor severe acute respiratory syndrome (SARS) and Middle East respiratory syndrome (MERS) virus.^1^ These viruses are enveloped, single‐stranded, positive‐sense RNA viruses that belong to the family Coronaviridae. The virus causing COVID‐19 (SARS‐CoV‐2) enters into the body via angiotensin‐converting enzyme (ACE‐2) receptors and can cause bronchial epithelial damage, alveolar interstitial hyperplasia, and fibrosis, which can contribute to future dyspnea.^2^

Dyspnea is one of the most prevalent symptomatic manifestations of COVID‐19. It is currently unknown what percentage of patients will experience dyspnea after COVID‐19, and what might be the possible reasons for such post‐COVID dyspnea. But there are pieces of evidence that dyspnea is significantly correlated with admission to intensive care units and subsequent patient mortality.^3^, ^4^

Severe dyspnea indicates that there is the involvement of more lung lobes.^5^, ^6^ During acute illness, the dyspnea can be due to pneumonia, acute respiratory distress syndrome (ARDS), or to some extent acute pulmonary embolism.^7^ Many patients recover from the initial respiratory symptoms after the acute presentation of COVID‐19.

On the other hand, a considerable number of discharged patients after recovering from acute illness experience post‐COVID‐19 dyspnea.^8^ It is unknown which of these patients might experience dyspnea and whether it is predictable. The focus of this study is to find the prevalence and the associated risk factors for having dyspnea 2 months after recovering from an acute illness due to COVID‐19.

## 2 METHODS

### 2.1 Study design and participant selection

The retrospective cross‐sectional study was performed on the patients hospitalized at Dhaka Medical College and Hospital (abbreviated DMCH) located in Bangladesh from July to August 2020. The patients in this study were interviewed, 2 months post‐COVID‐19, via telephone and were asked a set series of questions, and their responses were recorded. All patients who met the following criteria were contacted for a follow‐up: admitted with moderate to severe symptoms of COVID‐19 according to the national guideline; a positive report of real‐time reverse transcriptase‐polymerase chain reaction (RT‐PCR) for SARS‐CoV‐2; and patients who met the WHO criteria for discharge from hospital (no fever for 3 days and improvement of other symptoms).^9^

### 2.2 Case definition

Regarding the initial evaluation of dyspnea among enrolled patients, they are asked the following question during the admission procedure: “have you experienced respiratory distress since the appearance of your first symptoms?” Furthermore, patients' oxygen saturation level was measured at the time of admission. Patients with an oxygen saturation level (SpO_2_) of ≤93% were considered hypoxic according to “National Guidelines on Clinical Management of COVID‐19” in Bangladesh.^10^ Patients who answered “yes” to the question and who were hypoxic at the time of admission were considered for the study, and patients who had given informed written consent were followed up 2 months later.

### 2.3 Data collection and evaluation of dyspnea after 2 months

All the demographic and clinical data of the patients were collected after hospitalization. Patients' demographics and clinical data included age, gender, smoking history, admission oxygen saturation level (SPO_2_), any prevailing comorbid condition, and common laboratory parameters such as hemoglobin percentage (Hb%), neutrophil percentage, C‐reactive protein (CRP), serum ferritin, and D‐dimer level.

**FIGURE 1:**
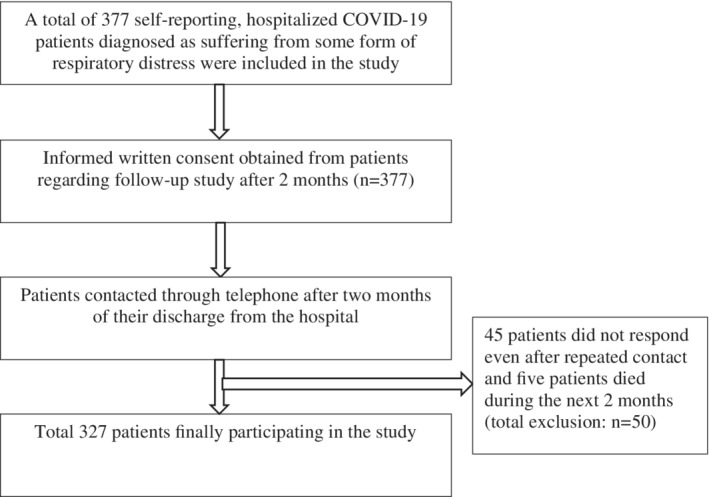
Schematic diagram of participant selection

All patients were then contacted 2 months after their hospital discharge through mobile phone (number provided during hospital admission). In the case of nonresponders, a text message was sent to their mobile phone after trying to contact them for three consecutive days. Participants failing to report for telephonic interviews were then ultimately excluded from the study. A schematic diagram entailing selection of study participants is illustrated in Figure 1.

The questionnaire was standardized to see potential correlations between COVID‐19 and prolonged dyspnea after recovery. They were evaluated using a modified 12‐item dyspnea questionnaire developed by Yorke et al.^11^ In the original questionnaire, patients were categorized into four categories based on their responses: no dyspnea, mild, moderate, and severe dyspnea. In this modified version, patients were asked regarding their subjective perception of dyspnea, and finally were categorized, taking into consideration their responses to the original 12‐item questionnaire, into the following categories: no dyspnea/fully recovered, present but improving, same as during hospital admission, and worsening of initial symptoms.

### 2.4 Statistical analysis

A chi‐square test was used to find the association between groups of patients. Continuous variables were expressed as median and interquartile ranges, and categorical variables were expressed as frequency (n) and percentages (%). For all statistical analyses, a *P* value of <.05% was considered statistically significant. Statistical analysis was carried out in R‐programming language.

### 2.5 Ethical consideration

Ethical permission was obtained from Dhaka Medical College ethical review board (ERC‐DMC/ECC/2020/91; 18‐05‐2020), and informed written consent was obtained from patients or their attendants before proceeding with the study.

## 3 RESULTS

The study was conducted on male and female patients with an average age of 50 years (range, 35‐70 years) of different lifestyles, habits, and with different underlying conditions. The demographic and clinical data of this study are summarized in Table 1.

**TABLE 1:** Demographic and presenting clinical characteristics of the study participants

Parameters	Frequency (%) (n = 327)
Male	214 (66%)
Female	113 (34%)
(Average) age in years	50 (37, 63)
Smoker	59 (18%)
SpO_2_ (%) at admission	88.0 (85.0, 91.0)
Hypertension	147 (45%)
Diabetes mellitus	134 (41%)
Ischemic heart disease	56 (17%)
Asthma	44 (13%)
Chronic obstructive pulmonary disease	21 (6.4%)
Chronic kidney disease	19 (5.8%)
Malignancy	2 (0.6%)

The results indicated that males accounted for more cases of COVID‐19 than females. Most of the patients were hypoxic during admission at the hospital for COVID‐19, showing an average oxygen saturation level (SpO_2_) of 88.0, and this is statistically significant with (prevalence of) dyspnea (*P* = .030). The most common comorbidities or underlying risk factors among the COVID‐19 patients were hypertension (HTN) (45%) and diabetes mellitus (DM) (41%), followed by ischemic heart disease (IHD) (17%) and asthma (13%).

The results obtained in the study revealed that out of a total of 327 patients, recovery from dyspnea was observed in 66.4% (n = 217) patients, and 4.6% (n = 15) patients reported the same as they experienced during COVID‐19 illness; 27.5% (n = 90) patients felt better than that of earlier exposure of COVID‐19 but not fully recovered, and only 1.5% (n = 5) patients complained further worsening compared with the previous dyspnea status. That means a total of 34% of participants experienced dyspnea, after 2 months of hospital discharge to some extent.

Evidence of significant covariates for dyspnea of the study participants is depicted in Table 2. The patients’ SpO_2_, D‐dimer, ferritin, and neutrophils count were measured on the day of admission to the hospital. All *P* values were derived by Pearson's chi‐square test. It shows that there is a significant association between D‐dimer (*P* value <.001) and serum ferritin (*P* value <.006) with dyspnea (Table 2).

**TABLE 2:** Evidence of significant covariates for dyspnea

Parameters	Deteriorated (n = 5)	Constant (n = 15)	Improved (n = 90)	Recovered (n = 217)	*P* value
Age	47 (46, 58)	65 (52, 70)	53 (42, 62)	47 (35, 57)	.001*
Male	3 (60%)	10 (67%)	68 (76%)	133 (61%)	.10
Female	2 (40%)	5 (33%)	22 (24%)	84 (39%)	.10
**Smoker**	**1 (20%)**	**5 (33%)**	**24 (27%)**	**29 (13%)**	.012*
HTN	3 (60%)	6 (40%)	47 (52%)	91 (42%)	.3
DM	4 (80%)	7 (47%)	36 (40%)	87 (40%)	.4
Asthma	1 (20%)	2 (13%)	29 (13%)	12 (13%)	.9
IHD	0 (0%)	4 (27%)	19 (21%)	33 (15%)	.3
**COPD**	**0 (0%)**	**2 (13%)**	**11 (12%)**	**8 (3.7%)**	.021*
CKD	0 (0%)	1 (6.7%)	3 (3.3%)	15 (6.9%)	.6
**SpO**_**2**_ **(%)**	**87.0 (86.0, 89.0)**	**86.0 (84.5, 89.0)**	**87.0 (85.0, 90.0)**	**89.0 (86.0, 92.0)**	.030*
**Serum ferritin (μgm/L)**	**864 (762, 988)**	**517 (384, 1039)**	**406 (170, 680)**	**327 (176, 639)**	.006*
**D‐dimer (μgm/L)**	**1.79 (1.30, 2.00)**	**1.50 (0.81, 2.02)**	**0.96 (0.50, 1.40)**	**0.63 (0.43, 1.12)**	.001*
Neutrophils (%)	3.6 (2.8, 6.2)	7.1 (2.7, 8.9)	5.6 (3.5, 8.8)	4.8 (3.2, 7.0)	.4

Abbreviations: CKD, chronic kidney disease; COPD, chronic obstructive pulmonary disease; DM, diabetes mellitus; HTN, hypertension; IHD, ischemic heart disease.

* *p* value less than 0.05 was considered significant.

The bold values are indicates significance value <0.05.

Smoking was also found to be significantly associated in Pearson's chi‐square test, with the grading of changes in dyspnea (*P* = .012). The percentage of smokers was much less in the deteriorated group (only 1), and there were more smokers in the recovered (n = 29, 13%) and improved (n = 24, 27%) groups. A significant statistical relationship was observed between SpO_2_ level (*P* = .030) and the presence of COPD (*P* = .021) across groups (Table 2).

## 4 DISCUSSION

The main reason for many patients seeking medical care with COVID‐19 is the recurrence of dyspnea. Patients were often panicked about seeking treatment due to the social stigma regarding the COVID‐19 outbreak. Dyspnea was one of the main concerns and stimuli that prompted a significant number of patients in Bangladesh to get admitted for the treatment of COVID‐19.^12^, ^13^ Our primary concern was to evaluate what proportion of these patients experienced dyspnea in the long run, after being discharged from the hospitals.

A total of 34% of participants experienced dyspnea after 2 months of hospital discharge to some extent. In a similar study, it was found that among 488 participants who completed a survey 60 days after hospital discharge, 159 patients (~32.6%) reported cardiopulmonary symptoms (such as cough and dyspnea).^14^ According to a study conducted in Italy on 143 patients, it was observed that a high proportion of individuals (43.4%) reported dyspnea in the follow‐up post‐acute care assessment.^7^ Among 150 patients with noncritical COVID‐19, 36.7% patients reported dyspnea at day 30 and 30% patients reported dyspnea at day 60 in a follow‐up study conducted in France from 17 March to 3 June 2020.^15^ Furthermore, in another phone‐based survey of 120 patients (96 patients, who had not been in the ICU, and 24 patients, who had been in the ICU), 42% patients suffered from dyspnea and comparisons between ICU and non‐ICU patients showed no statistically significant differences regarding dyspnea.^16^

At the beginning of the pandemic, it was well documented that the presence of comorbid conditions is an important risk factor for unfavorable outcomes. One study showed that after adjusting for age and smoking status, COPD (hazards ratio [HR] 2.681, 95% confidence interval [95% CI] 1.424‐5.048), diabetes (HR 1.59, 95% CI 1.03‐2.45), hypertension (HR 1.58, 95% CI 1.07‐2.32), and malignancy (HR 3.50, 95% CI 1.60‐7.64) were risk factors of reaching to the composite endpoints.^16^ The prevalence of dyspnea post‐COVID failed to show any statistically significant associations with diabetes, hypertension, asthma, IHDs, and chronic kidney disease (CKD). But COPD did show significant associations. Serum ferritin, D‐dimer, and tachypnea with or without respiratory crackles predict whether patients with COVID‐19 will require ICU admission.^17^ But any association between long COVID symptoms or so‐called “long‐haulers” symptoms with basic demographic characteristics, for example, age, sex body weight, initial SpO_2_, as well as few affordable laboratory parameters, for example, serum ferritin and D‐dimer are not well studied.

Our data resemble the hypothesis of the “smoker's paradox” present in COVID‐19 as proposed by several experts.^18^, ^19^, ^20^ As in our case, the number of smokers was surprisingly lower among COVID‐19 patients, and smoking seems to be a protective factor against dyspnea in long‐term follow‐ups, which has not yet been established by enough scientific data. Several mechanisms have been proposed for smoking being a protective factor in COVID 19 patients.^18^, ^19^, ^20^ One of such mechanisms is through the regulation of ACE2. It is well known that ACE2 provides overall protective effects on the lungs, and SARS‐CoV‐2 binds with this enzyme as a receptor and downregulates it. There are conflicting reports about the upregulation and downregulation of ACE2 by nicotine.^20^, ^21^, ^22^ It is too early to reach a concrete conclusion about the apparent protective effects of smoking in dyspnea, and further clinical studies with acceptable sample participants are required. On the other hand, COPD patients are always hypoxic, and while they have COVID‐19 they become more distressed, and there may be a great role of social stigma. Overall, they became hopeless, but after recovery, they feel better in comparison to the hospital period.

Nevertheless, the presence of several biases cannot be ruled out. The reason for such a result may be since those who had COPD had a decreased SpO_2_ on admission and had been exposed to vigorous restriction of smoking. This would lead to improvement in dyspnea in the subsequent follow‐ups, and the percentage of improvement would be noticed greatly in comparison with a non‐smoker, non‐COPD patients. So, it seems that this low incidence of dyspnea among smokers is not because of smoking rather it is because of cessation of smoking due to restricted hospitalized environment.

It is well known that at the beginning of the pandemic, there were enormous social media stigmas that created panic and stress among people hindering them from seeking treatment until they developed severe dyspnea and hypoxia.^16^, ^17^ This study shows that 19 of 21 participants (~90.5%) with a history of concomitant COPD felt significant improvement after 2 months of COVID‐19 and none of the participants experienced deterioration of respiratory distress after discharge from the hospital. A possible explanation of such seemingly unusual telephonic patient reply is that the patients being discharged from the hospital started feeling much more protected, less stigmatized, and more socially supported compared with their feelings of “during COVID time”, which brought a sense of mental peace for them.

There are several limitations to our study. First, the sample size was smaller compared with several other recent studies. This is because we did not receive any funding for this study and hence were restricted in terms of manpower and other logistics. Furthermore, we interviewed all patients through a telephonic conversation rather than face‐to‐face interview. This may be a source of business, as the interviewer had to rely on the responses of the patients and could not judge the validity of their responses properly.

## 5 CONCLUSION

In this study, a significant proportion of patients were found to be still suffering from dyspnea after their discharge from the hospital. While the number of patients enrolled in this study was low, these findings can still be useful for the clinicians to recognize the high prevalence of dyspnea in the post‐acute setting and to predict long‐term complications of hospitalized patients. This recognition may help to guide the treatment and management of dyspnea in post‐COVID patients. Furthermore, policy makers should take note of our findings and plan for long‐term management of post‐COVID complications through the implementation of follow‐up clinics and rehabilitation programs for affected individuals.

## FUNDING

This research received no specific grant from any funding agency in the public, commercial, or not‐for‐profit sectors.

## CONFLICT OF INTEREST

Nothing to disclose.

## AUTHOR CONTRIBUTIONS

Conceptualization: Md. Khairul Islam, Md. Robed Amin, Md. Maruf Ahmed Molla, Mohammad Faisal Hossain, Ayesha Sikder, Md Gias Uddin

Data Curation: Md. Mohiuddin Sharif, Pratyay Hasan, Fahima Sharmin Hossain

Methodology: Md. Maruf Ahmed Molla, Md. Khairul Islam, Md. Robed Amin, Mohammad Faisal Hossain, Ayesha Sikder, Md Gias Uddin

First Draft Preparation: Md. Khairul Islam, Md. Mohiuddin Sharif

Review and Editing: Md. Maruf Ahmed Molla, Pratyay Hasan, Fahima Sharmin Hossain

Supervision: Md. Khairul Islam, Md. Robed Amin, Ayesha Sikder, Mohammad Faisal Hossain

All authors have read and approved the final version of the manuscript.

Md. Maruf Ahmed Molla had full access to all of the data in this study and takes complete responsibility for the integrity of the data and the accuracy of the data analysis.

## TRANSPARENCY STATEMENT

Corresponding author MMAM guarantees that the manuscript is an honest, accurate, and transparent account of the study being reported; that no important aspects of the study have been omitted; and that any discrepancies from the study as planned (and, if relevant, registered) have been explained.

## Data Availability

The data that support the findings of this study are available on request from the corresponding author. The data are not publicly available due to privacy or ethical restrictions.
